# Hand-assisted retroperitoneoscopic versus standard laparoscopic donor nephrectomy: HARP-trial

**DOI:** 10.1186/1471-2482-10-11

**Published:** 2010-03-25

**Authors:** Leonienke FC Dols, Niels FM Kok, Turkan Terkivatan, TC Khe Tran, Frank CH d'Ancona, Johan F Langenhuijsen, Ingrid RAM zur borg, Ian PJ Alwayn, Mark P Hendriks, Ine M Dooper, Willem Weimar, Jan NM IJzermans

**Affiliations:** 1 Department of Surgery, Erasmus MC, Rotterdam, the Netherlands; 2 Department of Urology, Radboud University Nijmegen Medical Centre, Nijmegen, the Netherlands; 3 Department of Anaesthesia, Erasmus MC, Rotterdam, the Netherlands; 4 Department of Surgery, Dalhousie University, Halifax, Nova Scotia, Canada; 5 Department of Anaesthesia, Radboud University Nijmegen Medical Centre, Nijmegen, the Netherlands; 6 Department of Nephrology, Radboud University Nijmegen Medical Centre, Nijmegen, the Netherlands; 7 Department of Internal Medicine, Erasmus MC, Rotterdam, the Netherlands

## Abstract

**Background:**

Transplantation is the only treatment offering long-term benefit to patients with chronic kidney failure. Live donor nephrectomy is performed on healthy individuals who do not receive direct therapeutic benefit of the procedure themselves. In order to guarantee the donor's safety, it is important to optimise the surgical approach. Recently we demonstrated the benefit of laparoscopic nephrectomy experienced by the donor. However, this method is characterised by higher in hospital costs, longer operating times and it requires a well-trained surgeon. The hand-assisted retroperitoneoscopic technique may be an alternative to a complete laparoscopic, transperitoneal approach. The peritoneum remains intact and the risk of visceral injuries is reduced. Hand-assistance results in a faster procedure and a significantly reduced operating time. The feasibility of this method has been demonstrated recently, but as to date there are no data available advocating the use of one technique above the other.

**Methods/design:**

The HARP-trial is a multi-centre randomised controlled, single-blind trial. The study compares the hand-assisted retroperitoneoscopic approach with standard laparoscopic donor nephrectomy. The objective is to determine the best approach for live donor nephrectomy to optimise donor's safety and comfort while reducing donation related costs.

**Discussion:**

This study will contribute to the evidence on any benefits of hand-assisted retroperitoneoscopic versus standard laparoscopic donor nephrectomy.

**Trial Registration:**

Dutch Trial Register NTR1433

## Background

Transplantation is the only treatment offering long-term benefit to patients with chronic kidney failure. As the number of patients suffering end stage renal disease (ESRD) increases, the recruitment of more kidney donors is important [1]. Live kidney donation is the most realistic option to reduce donor shortage on short- and long-term. Increasing the number of donors will decrease the number of patients on the waiting list and consequently reduce patient's mortality. Implementation of live donation offers the possibility to transplant before the kidney disease reaches the terminal phase, necessitating dialysis. Thus, this so called pre-emptive transplantation may prevent unnecessary surgical interventions to establish dialysis (including costs and mortality) and dialysis related complications. In the last decade the number of non-related live kidney donations is rising. Among these donors are family and friends of the recipient, and even anonymous donors; the ethical basis for live kidney donation is altering. The looser the connection between the donor and recipient is, the less clear the profit for the donor is.

Live donor nephrectomy is performed on healthy individuals who do not receive direct therapeutic benefit of the procedure themselves. In order to guarantee safety for the donors, it is important to optimise the surgical approach. Recently we demonstrated the benefit of laparoscopic nephrectomy (LDN) to the donor. However, this method is characterised by higher in-hospital costs, longer operating times and requires a well-trained surgeon [2]. The hand-assisted retroperitoneoscopic technique (HARP-technique) may be an alternative to a complete laparoscopic, transperitoneal approach. The peritoneum remains intact and the risk of visceral injuries is reduced. The hand-assistance results in a faster procedure and a significantly reduced operating time [3-7]. The feasibility of this method has been demonstrated recently, but as to date studies advocating the use of one technique above the other are lacking. This randomised controlled trial compares the hand-assisted retroperitoneal approach to the current standard, the transperitoneal laparoscopic technique, to define the most optimal approach.

We recently proved that laparoscopic kidney donation is beneficial for the donor. In comparison to minimally invasive open techniques, laparoscopic kidney donation is associated with a better quality of life, less pain, shorter in hospital stay and earlier return to work. This method is expensive for the hospital, has a long operating time and requires an experienced, well-trained, surgeon [2,8,9]. Other studies showed a possibly increased rate of life threatening complications, such as injuries to the intestines and bleeding [10,11]. A surgical approach that is easier to learn and applicable in the majority of donors (i.e. selection of donors is not required) with similar benefits as the transperitoneal laparoscopic approach is warranted.

The hand-assisted retroperitoneoscopic approach may be a viable alternative. With this method the surgeon inserts his hand to create a retroperitoneal space, which is thereafter insufflated with gas. The peritoneum stays intact and tactile sensation remains. The chance of a complication to the intestines is very small. Furthermore, this technique is easier and quicker to learn for the surgeon than the laparoscopic approach. There is no randomized controlled trial comparing both techniques for the effectiveness [3-6].

## Methods/Design

### Study objective

To determine the best approach for live donor nephrectomy i.e. to optimise donor's safety and comfort while reducing donation related costs.

### Study design

The HARP-trial is a multi-centre, randomised controlled, single-blind trial. We have stratified per centre. The study started on July 24th 2008 and the duration of the inclusion will be approximately 3 years. The study compares hand-assisted retroperitoneoscopic donor nephrectomy and standard laparoscopic donor nephrectomy. In total 190 live kidney donors will be included in the study. Approval of the medical ethical committee of both centres was obtained.

Randomisation will take place after endotracheal intubation, by means of telephonic consultation of the study coordinator. A computer generated randomisation list with hidden block size is made for each centre by the statistician. The donor and the physicians involved in the postoperative period are blinded to the surgical technique until one year after donor nephrectomy. The operating theatre is not accessible for people who do not join the operating team. An independent surgeon evaluates the donor on the outpatient clinic before operation. As the extraction incision is similar in both techniques, we did not attempt to cover the wounds with a standard pattern of bandages [12]. All donors fill out the questionnaires until one year after donation, the Short-form 36 (SF-36), Euroqol (EQ-5D), Visual Analogue Scale (VAS) and a questionnaire about work and homecare.

### Patient selection

All, properly Dutch speaking, live kidney donors who are medically capable of donating their left kidney can be included in the HARP-trial. Informed consent is mandatory. All types of live kidney donors can participate in the study, i.e. related, unrelated, altruistic and cross-over live kidney donors.

Exclusion criteria are a history of kidney surgery or adrenal gland surgery on the left side.

All potential donors are informed about the study at the outpatient clinic. For further information the patient can refer to the research fellow, transplant surgeon, or the independent physician. If a patient does not sign the informed consent form, the patient is not included in the study and therefore will be operated via the standard protocol. A live kidney donor can always withdraw his or her consent at anytime during the study.

### Hypothesis

The left-sided hand-assisted retroperitoneal approach will lead, with a similar or better quality of life, to fewer complications, and reduced operating times and costs.

### Study Questions

Primary Question: Does left-sided hand-assisted retroperitoneoscopic donor nephrectomy lead to a similar or better quality of life as compared to left-sided transperitoneal laparoscopic donor nephrectomy?

Secondary Question: Is the retroperitoneoscopic technique safer (conversions and complications) than the transperitoneal technique?

Other secondary endpoints: pain perception, return to work, operation time, cost-effectiveness from both a societal and health care perspectives (costs per quality adjusted life year).

### Pilot study

The Erasmus Medical Centre Rotterdam harbours one of the largest European live kidney donor transplantation programs. On a yearly basis 75 to a 100 laparoscopic donor nephrectomies are performed. Our strategy is to improve the results of live kidney donation by optimizing the surgical technique with decreased complication rates and costs. The infrastructure, raised in earlier studies, led to the formation of a multidisciplinary team, with a high standard of care and surgery.

In the previous study on live kidney donation running at our centre we compared the laparoscopic technique to the mini-incision muscle-splitting technique [2]. Laparoscopic donor nephrectomy resulted in early recovery, less fatigue and a better quality of life. However, the laparoscopic technique was more expensive from a hospital point of view and demanded more experience from the surgeon.

A pilot study with 60 live kidney donors showed the feasibility of the retroperitoneal hand-assisted approach [3]. Even in this small group operating time was significantly reduced. It seems feasible to expand the surgical armamentarium with this technique. However, we first have to demonstrate similar or better quality of life in comparison with the laparoscopic technique.

### Surgical intervention

Both procedures were performed with the donor placed in right-decubitus position. LDN was performed as described earlier [8]. Shortly, a 10-mm trocar was introduced under direct vision. The abdomen was insufflated carbon dioxide to 12 cm H_2_O pressure. A 30° video endoscope and 3 to 4 additional trocars were introduced. The colon was mobilized and displaced medially. Opening of the renal capsule and division of the perirenal fat was facilitated using an ultrasonic device (Ultracision, Ethicon, Cincinnati, USA). After identification and careful dissection of the ureter, the renal artery and the renal vein, a pfannenstiel incision was made. An endobag (Endocatch, US surgical, Norwalk, USA) was introduced into the abdomen. The ureter was clipped distally and divided. The renal artery and vein were divided using an endoscopic stapler and the kidney was placed in the endobag and extracted through the pfannenstiel incision.

In HARP we started with a 7-10 cm pfannenstiel incision [3]. After blunt dissection to create a retroperitoneal space, the Gelport (Applied Medical, Rancho Santa Margarita, California, USA) was inserted. Blunt introduction of the first trocar between the iliac crest and the handport was guided by the operator's hand inside the abdomen. CO_2 _was insufflated retroperitoneally to 12 cm H_2_O pressure. Two other 10-12 mm trocars, respectively just outside the midline inferior to the costal margin and in the flank, were inserted to create a triangular shape. For dissection the aforementioned ultrasonic device was used. Dissection of the kidney and renal vessels was similar to transperitoneal donor nephrectomy but with hand-assistance and from a slightly different angle. The kidney was removed manually.

### Outcome measures

Primary outcome: Physical functioning as a measure for quality of life one month after donor nephrectomy. This is one of the dimensions of the standardized SF-36 questionnaire. Physical functioning is measured by means of the complaints with daily physical activities, like walking stairs, carrying grocery bags, walking 500 meter, etc. The SF-36 is in our opinion a suitable parameter to measure post-operative recovery [13].

Secondary outcome: Direct costs (costs for the hospital). Other secondary endpoints: conversion to open surgery, complications, pain perception, work resumption, other dimensions of quality of life (SF-36), cost-effectiveness from hospital and health care perspective.

### Costs-effectiveness

In this effectiveness analysis the effect of the surgical procedure on quality of life of the donor and the costs for the hospital are the most important outcome measures. The donor is the central point of interest; therefore we chose quality of life as primary endpoint for the power calculation. Saving money, but with a worse quality of life for the donors is indeed not relevant.

### Power calculation

A difference of 7.5 point in physical functioning (SF-36) is a relevant difference. In international literature, a five point difference is the minimal clinically relevant difference [13]. In our previous study, reviewers thought this difference of five points was too small. Ten points is a big difference, but in our previous study this difference was observed after one month. A difference of 7.5 is in the middle of these data. Physical functioning is expressed on a scale of zero till hundred. Zero means a very limited function and hundred means an unrestrained function. With a measured standard deviation of 18.4 points (reference for left-sided kidney donation in the last five years), an alpha of 0.05, and a beta of 0.2, 95 donors have to be enrolled in both groups (Altman, Practical statistics for medical research, Chapman&Hall/CRC, 1991). Hereby we test two-sided, because the hand-assisted technique could be similar or even better than the laparoscopic technique. All data will be analysed according to the 'intention to treat' principle. Costs and effectiveness will be combined with the Euroqol 5-D to express the difference between both techniques in quality adjusted life years (QALYs).

### Treatment of participating live kidney donors

The donor is prehydrated with intravenous crystalloids before operation. On the morning of surgery antithrombotic stockings are given. The anaesthetist uses a standard protocol for live kidney donation anaesthesia (remifentanyl and propofol), intravenous policy, and respiration. One hour after the beginning of the operation, 20 mg mannitol is infused. During the operation the research-fellow notes warm-ischemia time, blood loss, and complications. Postoperative pain medication is measured through a Patient Controlled Analgesic (PCA; morphine) device. If the patient does not use the PCA for 6 hours, the PCA is stopped. The dosage regimen is registered. Patients can be discharged when they meet with the following criteria:

- The donor tolerates a normal diet

- The donor is mobile (is able to walk stairs)

- The donor is adequate and oriented

- The donor does not use intravenous medication

All live kidney donors will be seen at the outpatient clinic after four weeks, three months and yearly thereafter. All donors are asked to fill out different questionnaires; we measure pain and nausea scores (VAS-score), quality of life (SF-36, EQ-5D), and a questionnaire on work and homecare (Table 1).

**Table 1:** Time schedule for filling out the questionnaires

Time of evaluation	VAS	EuroQol	SF-36	Work and homecare
**Preoperative**	X	X	X	X
**Postoperative**				
**Day 0**	X			
**Day 1**	X			
**Day 2**	X			
**Day 3**	X	X		
**Week 1**	X	X		X
**Week 2**	X	X		X
**Week 4**		X	X	X
**Week 6**			X	X
**Week 8**			X	X
**Month 3**		X	X	X
**Month 6**		X	X	X
**Month 12**		X	X	X

### Unexpected events

The live kidney donor is informed at the outpatient clinic on the background of the study, possible complications and the chance of conversion to an open approach. After both operations the donor may wake up having an extra scar caudal to the costal margin if the operation is converted to an open approach.

If a donor has post-operative pain or discomfort of the bandages, they can be removed. All documentation will be marked with the HARP-trial logo. Information about the operation technique will be sealed in an envelope in the medical file. In case of unexpected events this envelope may be opened. Unexpected events are reported to the responsible physician and the study coordinator.

### Access to personal data

All personal data are coded into numbers (1 to 190). The coordinating investigator and the principle investigator are the only ones who have access to the coding system. All data are imported in a database, which is managed by the coordinating investigator. At the end of the trial all data are analysed together with the trial statistician.

## Discussion

The hand-assisted retroperitoneoscopic technique may be an alternative to a complete laparoscopic, transperitoneal approach. The feasibility of this method has been demonstrated recently, but as to date there are limited data available advocating the use of one technique above the other [3-7]. This randomised controlled trial compares the hand-assisted retroperitoneal approach to the current standard, the transperitoneal laparoscopic technique to define the most optimal approach.

In comparison to minimally invasive open techniques, laparoscopic kidney donation is associated with a better quality of life, less pain, shorter in-hospital stay and earlier return to work. This method is time consuming, leading to high hospital costs, and requires an experienced surgeon. Other studies showed a possibly increased rate of major, life threatening, complications, such as injuries to the intestines and bleeding [10].

The hand-assisted retroperitoneoscopic approach may be a viable alternative. The chance of a complication to the intestines is very small, and hand-assistance could be beneficial for the control of bleedings. There is no randomized controlled trial comparing both techniques. Only three small studies compare left-sided hand-assisted retroperitoneoscopic with laparoscopic donor nephrectomy, but only with respect to clinical parameters [3-5,7]. Wadstrom *et al *compared the LDN (n = 11), hand-assisted laparoscopic technique (HALS) (n = 14), and the HARP (n = 18). The operation time with the HARP was significantly shorter than the LDN (141 vs. 270 min, p < 0.01) [7]. Sundqvist *et al *performed a prospective study, comparing HARP (n= 11), LDN (n= 14) and open donor nephrectomy (n= 11). Hand-assisted retroperitoneoscopic donor nephrectomy had a significantly shorter operation time compared to LDN (145 min vs 218 min, p < 0.05) [5]. Gjertsen *et al *performed a retrospective study, comparing HARP (n= 11), LDN (n= 15) and open donor nephrectomy (n= 25). Reduced operation time was observed for the HARP group compared with the LDN (166 min vs 244 min) [4]. Dols *et al *performed a prospective study, comparing 20 left-sided HARP procedures and 40 left-sided LDNs. Median operation time and WIT were shorter in HARP (180 vs. 225 min, p = 0.002 and 3 vs. 5 min, p = 0.007 respectively) [3].

All other studies only described surgical outcome, and did not address quality of life of live kidney donors. Our main point of interest is to know whether the donors operated on with the hand-assisted retroperitoneal technique have a good quality of life afterwards. Therefore we will perform this randomised controlled trial comparing laparoscopic to hand-assisted retroperitoneoscopic technique, with physical function as a primary outcome.

## Competing interests

The authors declare that they have no competing interests.

## Authors' contributions

LFCD and NFMK drafted the manuscript. JNMIJ co-authored the writing of the manuscript. All other authors participated in the design of the study during several meetings and are local investigators at participating centres. All authors have read and approved the final manuscript.

## Pre-publication history

The pre-publication history for this paper can be accessed here:

http://www.biomedcentral.com/1471-2482/10/11/prepub
